# Editorial: Open Dialogue around the world – implementation, outcomes, experiences, and perspectives

**DOI:** 10.3389/fpsyg.2025.1621237

**Published:** 2025-06-05

**Authors:** Raffaella Pocobello, Jaakko Seikkula, Rob Saunders, David Mosse, Sebastian von Peter

**Affiliations:** ^1^Institute of Cognitive Sciences and Technologies, Department of Human and Social Sciences, Cultural Heritage, National Research Council, Rome, Italy; ^2^Department of Psychology, University of Jyväskylä, Jyväskylä, Finland; ^3^CORE Data Lab, Centre for Outcomes Research and Effectiveness, University College London, London, United Kingdom; ^4^Centre for Anthropology and Mental Health Research in Action, SOAS University of London, London, United Kingdom; ^5^Brandenburg Medical School, Immanuel Klinik Ruedersdorf, Centre of Mental Health - Psychiatry and Psychotherapy, Neuruppin, Germany

**Keywords:** Open Dialogue, peer supported Open Dialogue, evaluation, implementation, outcomes

Open Dialogue (OD) is receiving increased interest across mental health systems worldwide, inspiring practitioners, researchers, and policymakers to explore dialogical alternatives to conventional psychiatric models. Originally developed within the Finnish mental health system, OD is a systemic approach to care based on seven principles—five concerning the organization of services (immediate help, social network perspective, flexibility and mobility, psychological continuity, and responsibility), and two reflecting a dialogical way of being with service users and their networks (tolerance of uncertainty and dialogism). While previous studies have outlined the theoretical foundations and reported promising outcomes of OD, much remains to be understood about how the approach is translated into practice as it encounters varied professional cultures, institutional logics, and system-level constraints.

This Research Topic builds on the questions raised in our introductory paper (Mosse, Pocobello, et al.) and brings together 24 contributions from 105 authors, offering a multifaceted overview of current research and implementation efforts in OD. Through empirical studies, conceptual analyses, and methodological developments, the collection explores the opportunities and tensions that emerge as OD is applied in new settings and reinterpreted through diverse local experiences.

## Implementing Open Dialogue in different contexts

The first section of the Research Topic examines how Open Dialogue has been implemented across different countries, reflecting a wide range of stages in its adoption and integration. Drawing on data from 24 countries, the HOPEnDialogue international survey conducted by Pocobello, Camilli, Ridente, et al. documents the growing global presence of OD, while also revealing considerable diversity in how its core elements are applied. Some services report high fidelity to the original model developed in Western Lapland, while others follow OD-inspired practices that have yet to incorporate key principles such as immediate response, network involvement, and continuity of care. This variability illustrates both the adaptability of the approach and the challenges of sustaining its essential values across diverse settings.

Starting with the data collected through the HOPEnDialogue survey, Heumann et al. focused on analyzing the implementation of OD in Germany, a country where, despite hundreds of professionals being trained in OD since 2007 and more than 40 services practicing the approach in the year of the study, several obstacles were observed, such as the fragmentation of the system of care. Additional expert interviews suggest that the structure of the services, as well as specific features the German mental health care system, are likely to underlie these barriers. These findings highlight the importance of considering structural and systemic issues alongside training efforts to enhance successful OD implementation.

In Vermont, United States, Alpern et al. explored organizational challenges related to the implementation of OD-informed practice through anticipation dialogues—a dialogical practice that encourages participants to envision a positive future scenario. Beyond proposing anticipation dialogue as a research tool, the authors identified key dilemmas, including tensions between systemic uncertainty experienced by staff and the need for flexible, inclusive, and non-hierarchical approaches to support dialogic practice. The authors suggest that achieving a sustainable integration of Open Dialogue requires clear structural support and leadership commitment.

In Spain, where OD has been introduced in the last few years, Parrabera-Garcia et al. conducted a preliminary evaluation regarding training, experience, access to materials and events, and perceived needs in OD implementation. The survey revealed a lack of trained professionals as well as insufficient training hours and limited access to resources, underscoring an urgent need for enhanced local training initiatives and translated materials to support the delivery of OD.

In contrast with the Spanish bottom-up request, in South Korea OD has been introduced through a top-down initiative by the Ministry of Health and Welfare as part of a project to support the dissemination of WHO QualityRights-based services. A mixed-method survey by Cho et al., aimed at investigating the experiences of professionals, highlighted some perceived challenges and limitations, and provided practical recommendations on how to better align OD training and implementation guidelines with local cultural and systemic contexts.

Using a different methodology (i.e., a focus group), Skourteli et al. explored similar research questions in an action-research project conducted in Greece. Their study traces the implementation journey of OD in a Day Center for Psychosocial Rehabilitation in Athens, highlighting the challenges faced by mental health professionals and their contextualization within the local organizations and culture. This approach provides valuable insights into how local organizational cultures can influence the adoption of OD practices.

Klatt et al. further explore this theme by reflecting on the development of an initiative in Germany grounded in grassroots democratic values and a shared intention for change. Their account suggests that such organizational characteristics may support the integration of dialogical principles, particularly within a community of practice composed largely of young professionals. The authors also point to dissonance as a potentially productive element in addressing crisis and institutional transformation.

Within the same national context, von Peter et al. offer further insights based on data from an implementation study that struggled to retain practitioners engaged with OD. The authors argue that dynamics related to power and professional identity lie at the core of potential failures in OD adoption, raising thought-provoking questions to inform future implementation strategies.

## Peer support, training, and professional reflections in Open Dialogue services

This section brings together studies that examine how Open Dialogue services are shaped by practices of peer support, professional training, and team reflection. These studies explore the development of participatory models, the articulation of core principles, and the challenges involved in communicating and embodying dialogical values within services.

Chmielowska et al. present a viewpoint on the importance of developing an OD model grounded in peer support and shared decision-making. Co-authored by individuals with lived experience of mental health issues, clinicians, and researchers, the paper offers a dialogical reflection on how values such as equality, transparency, and co-responsibility can shape team dynamics, supervision, and decision-making processes. It provides insights into the research and training needed to establish such a framework, while also acknowledging the tensions and uncertainties involved in co-producing a Peer-Supported Open Dialogue (POD) service.

On the same topic, Hendy et al. identified key principles that they described as foundational to Peer-Supported Open Dialogue. In their conceptual analysis, the authors suggest that defining these specific principles also has practical implications for the development of POD services, particularly in areas such as training, supervision, recruitment, and role specification. Their proposal of 10 evolving principles—including mutuality, attention to power, and dialogical responsibility—aims to support ongoing reflection and collective learning within teams, rather than setting fixed standards.

A qualitative study by Lorenz-Artz et al. explored how to better present and explain POD to professionals who have not received the POD training. Based on interviews with practitioners working in the Netherlands, the authors identified four key themes, including the use of metaphors, positioning of the professional, handling uncertainty, and the importance of embodying dialogical principles. Insights from interviews with POD professionals led to valuable recommendations, which can inform the development of further guidance for professionals unfamiliar with POD. Notably, the study emphasizes that communicating the essence of OD requires more than technical explanation—it demands engaging with its values and experiential qualities.

Reflections on training are also presented by Thorley et al., who contribute a dialogical conversation they describe as “part (poly)-auto-ethnography and part perspective.” Drawing on their personal experiences as trainers in four different countries, the authors also invite readers into the dialogue, encouraging them to pause and reflect on their own thoughts and reactions as they engage with their writing. Their text explores the uncertainties, challenges, and moments of growth encountered in delivering OD training, aiming not to offer definitive answers but to remain open to multiple voices and meanings—reflecting the very spirit of dialogical practice.

## Experiencing Open Dialogue as a therapeutic process

Several papers in the Research Topic focus on the transformations associated with participating in network meetings, from the perspectives of the client, family members, and practitioners. These studies highlight how dialogical encounters can shape both therapeutic outcomes and professional identities, while also suggesting methodological tools to deepen understanding of these processes.

van Dieren and Clavero investigated the impact of reflective conversations on both the inner and outer dialogues of all participants. Beyond describing how reflections had an influence on the client and one of his family members, in their paper, the authors also propose the use of video-stimulated recall in social work, not only for research purposes but also as a tool that can further elicit new ideas and emotions. This method is presented as a dialogical tool in itself, offering practitioners a space to revisit conversations, recognize unspoken dynamics, and strengthen reflexivity.

Sidis et al. provided additional insights on the role of reflective conversations from the perspective of dialogical therapists. They present both the conceptualization of the reflective process and the concrete actions taken to facilitate it, offering insights into how OD professionals can cultivate and support this reflective attitude during network meetings. The authors explore how therapists maintain the balance between being present and facilitating reflection, pointing to the importance of emotional resonance, openness, and sustained attention to the evolving needs of the network.

Reflective processes were also explored by Lagogianni et al., who specifically focused on analyzing co-therapy dynamics during network meetings—a topic that, despite being central to the OD approach, has been scarcely investigated so far. By collecting information on the experiences of OD practitioners, the authors describe how co-therapy processes may develop and transform their own identity. The paper emphasizes that the therapeutic alliance extends beyond the client-therapist dyad, involving the relationship between co-therapists as a dynamic field of mutual adjustment, vulnerability, and growth.

On the same topic, Taylor et al. produced an auto-ethnographic account describing the changes they experienced as facilitators in network meetings over 2 years. Based on the positive impact felt by all three authors, they suggest that this transformation may lead to better outcomes in terms of staff retention, quality of life, and reduced burnout. Their narrative highlights how personal and professional boundaries are reshaped through dialogical work, and how training contexts themselves can become spaces of transformation and healing.

A different perspective is offered by Antoni, a physician who shares his 10-year experience applying OD in non-psychiatric settings. Reflecting on patients with physical symptoms potentially linked to psychological conditions, Antoni discusses the dilemmas other physicians may face when focusing on dialogue, their role as a “bridge between the biological and the psychic world,” and how dialogism can be applied in different medical fields.

## Outcomes of Open Dialogue interventions

While qualitative and process-oriented studies have provided valuable insights into the development and implementation of OD, promising results have also been achieved in the field of outcome studies.

Among these, a longitudinal study by Pocobello, Camilli, Alvarez-Monjaras, et al. represents one of the first efforts to systematically evaluate OD in routine public mental health care outside Finland. Conducted within Italian Mental Health Departments, the study followed 58 service users over one year and reported increased levels of satisfaction, improvements in psychological wellbeing and social functioning, a reduction in hospitalisations, and greater continuity in therapeutic relationships. These findings suggest that OD can be effectively integrated within community-based mental health systems committed to relational and recovery-oriented care.

In a brief research report, Tavares et al. describe a study conducted in the Alentejo region of Portugal that applied the same protocol to a smaller sample—seven service users and 21 network members. Despite the limited scale, the study contributes to the emerging international evidence on OD and underscores the importance of investigating outcomes across varied service contexts.

## Methodological developments in Open Dialogue research

Various research methodologies and tools have been explored in the Research Topic, highlighting both the opportunities they offer and the challenges they raise in the evaluation of OD.

Mosse, Baker, et al. discuss the contribution of anthropology—particularly ethnography—to understanding POD practices drawing on their work conducted in parallel with the ODDESSI study (Pilling et al., 2021). The authors reflect on how this discipline can contribute to and complement other forms of evidence on OD, such as randomized controlled trial (RCT) outcomes, while also raising important questions regarding researcher roles, positionality, and ethical dilemmas that may arise in immersive fieldwork.

Lotmore et al. report on the development of an adherence scale for use in the ODDESSI trial to assess whether the OD intervention was being applied as intended. After demonstrating the psychometric properties of the scale through analyses of network meeting audio recordings, their work resulted in a manual outlining the rating process and defining key elements of OD.

As a complementary initiative, Alvarez-Monjaras et al. developed and implemented a measure to assess various structural and organizational aspects of high-quality mental health services. The Community Mental Health Team Fidelity Scale (COM-FIDE), which consists of 25 items plus a seven-item OD addendum, was piloted to evaluate staff interviews, yielding encouraging preliminary psychometric results.

Finally, Fedosejevs et al. developed the Peer-supported Open Dialogue Attitude and Competence Inventory (PODACI), a self-report tool designed to assess trainees' preparedness after completing POD training, as well as the effectiveness of the training course. The PODACI, comprising 27 domains and 76 items, was developed using a four-round modified Delphi procedure but has not yet been undergone formal validation.

## Looking ahead

This Research Topic offers a broad and multifaceted overview of current research on Open Dialogue, while also pointing to important areas for further development. Advancing the theoretical understanding of how dialogical processes contribute to change remains a key priority—for clinical practice, for training and supervision, and for guiding future research.

As OD continues to diversify across settings and cultures, there is a growing need to revisit and elaborate its theoretical foundations, and to clarify what lies at the definitional core of this evolving field of practice. This also invites reflection on how our understanding of OD is shifting—whether as a clinical intervention, a paradigm for mental health care, or a broader movement for systemic and social change.

Greater involvement of service users and their social networks can play an important role in this process. Their perspectives offer insights that can deepen theoretical reflection and help ensure that evaluation remains connected to lived experience and everyday practice. Capturing outcomes and processes from their perspective is vital to understanding the ethical, relational, and transformative potential of OD.

Further consolidation of OD will require stronger empirical evidence, including results from randomized trials and large-scale international studies. Notably, there is currently a lack of studies on the use of medication within the context of Open Dialogue—an important aspect that should be addressed in future research. In parallel, more systematic implementation research is needed to understand how OD can be effectively and sustainably integrated into different service contexts. Hybrid studies that combine effectiveness and implementation outcomes may be particularly valuable, provided they adopt approaches consistent with the relational and dialogical principles of the model.

Another challenge lies in refining tools to assess fidelity and adherence to OD principles. Making these tools accessible and useful beyond research contexts—in training, supervision, and service development—could support ongoing quality improvement while maintaining coherence with the approach.

We hope this Research Topic will foster dialogue within the international OD community and encourage wider engagement with the OD approach. Continuing to build the evidence base remains essential to support its further development and broader adoption.
